# Schizandrin C regulates lipid metabolism and inflammation in liver fibrosis by NF-κB and p38/ERK MAPK signaling pathways

**DOI:** 10.3389/fphar.2023.1092151

**Published:** 2023-05-23

**Authors:** Panpan Chen, Rong Wang, Fangbin Liu, Shengnan Li, Yanqiu Gu, Lei Wang, Yongfang Yuan

**Affiliations:** ^1^ Department of Pharmacy, Shanghai Ninth People’s Hospital, Shanghai JiaoTong University School of Medicine, Shanghai, China; ^2^ School of Medicine, Shanghai University, Shanghai, China

**Keywords:** liver fibrosis, Schizandrin C, lipid metabolism, inflammation, NF-κB, p38/ERK MAPK

## Abstract

Liver fibrosis is considered a sustained wound healing response and metabolic syndrome, and its therapy is of great significance for chronic liver disease. Schizandrin C, as one lignan from hepatic protectant *Schisandra chinensis*, can depress the oxidative effect and lipid peroxidation, and protect against liver injury. In this study, C57BL/6J mice were used to estimate a liver fibrosis model by CCl_4_, and Schizandrin C exerted an anti-hepatic fibrosis effect, as evidenced by decreased alanine aminotransferase, aspartate aminotransferase and total bilirubin activities in serum, lower hydroxyproline content, recuperative structure and less collagen accumulation in the liver. In addition, Schizandrin C reduced the expressions of alpha-smooth muscle actin and type Ι collagen in the liver. *In vitro* experiments also revealed that Schizandrin C attenuated hepatic stellate cell activation in both LX-2 and HSC-T6 cells. Furthermore, lipidomics and quantitative real-time PCR analysis revealed that Schizandrin C regulated the lipid profile and related metabolic enzymes in the liver. In addition, the mRNA levels of inflammation factors were downregulated by Schizandrin C treatment, accompanied by lower protein levels of IκB-Kinase-β, nuclear factor kappa-B p65, and phospho-nuclear factor kappa-B p65. Finally, Schizandrin C inhibited the phosphorylation of p38 MAP kinase and extracellular signal-regulated protein kinase, which were activated in the CCl_4_ fibrotic liver. Taken together, Schizandrin C can regulate lipid metabolism and inflammation to ameliorate liver fibrosis by nuclear factor kappa-B and p38/ERK MAPK signaling pathways. These findings supported Schizandrin C as a potential drug for liver fibrosis.

## 1 Introduction

Liver fibrosis is considered a sustained wound healing response generated from chronic injury, which is accompanied by excessive deposition of extracellular matrix (ECM) (Massey et al., 2017). Many factors, such as alcohol abuse, chronic HCV infection, and nonalcoholic steatohepatitis (NASH), contribute to the occurrence and development of liver fibrosis (Bataller and Brenner, 2005). The degree of liver fibrosis is closely associated with liver function and is likely to further develop into liver cirrhosis and hepatocellular carcinoma (HCC) or result in chronic portal hypertension (Llovet et al., 2016; D'Amico et al., 2018; Pellicoro et al., 2014). Although some potential chemotherapeutic and bioactive substances for liver fibrosis have been developed, there remains a need for effective anti-fibrotic drugs in clinic (Fagone et al., 2016; Schuppan et al., 2018).

Lipids play a vital role in energy metabolism and cell signaling. In addition, lipids serve as the constituent of plasma membranes and lipid particles, including lipoproteins and extracellular vesicles (Meikle et al., 2021). Since the liver is the major organ of lipid metabolism, the imbalance of lipid metabolism is thought to be responsible for chronic liver damage, which may ultimately lead to liver fibrosis (Arain et al., 2017; Liu et al., 2022). Evidence has showed that lipid overload in hepatocytes drives hepatotoxicity and accelerates the progress of inflammation and fibrosis (Musso et al., 2018; Roehlen et al., 2020).

As a typical characteristic of chronic hepatic diseases, inflammation participates in and plays a crucial role in the occurrence and development of liver fibrosis (Ibrahim et al., 2018; Tu et al., 2021; Zhao et al., 2023). Persistent inflammation leads to tissue damage and promotes the release of pro-fibrotic cytokines, which can subsequently aggravate hepatocyte injury, activate hepatic stellate cells (HSCs), and trigger liver fibrosis (Seki and Schwabe, 2015; Tu et al., 2021).

Schizandrin C, one lignan from *Schisandra chinensis*, exerts an antioxidant effect, and inhibits lipid peroxidation and binding of CCl_4_ metabolites to lipids (Liu and Lesca, 1982; Lu and Liu, 1991). Recent research studies have reported that Schizandrin C protects against liver injury induced by acetaminophen and lithocholic acid (Jiang et al., 2015; Fan et al., 2019). However, the effect of Schizandrin C on liver fibrosis was unclear. In this study, C57BL/6J mice were used to build a liver fibrosis model by CCl_4_, and the anti-fibrosis effect of Schizandrin C was evaluated. In addition, the underlying mechanism of Schizandrin C anti-hepatic fibrosis-related lipid homeostasis and inflammation response was further investigated.

## 2 Materials and methods

### 2.1 Animals and *in vivo* experiments

**FIGURE 1:**
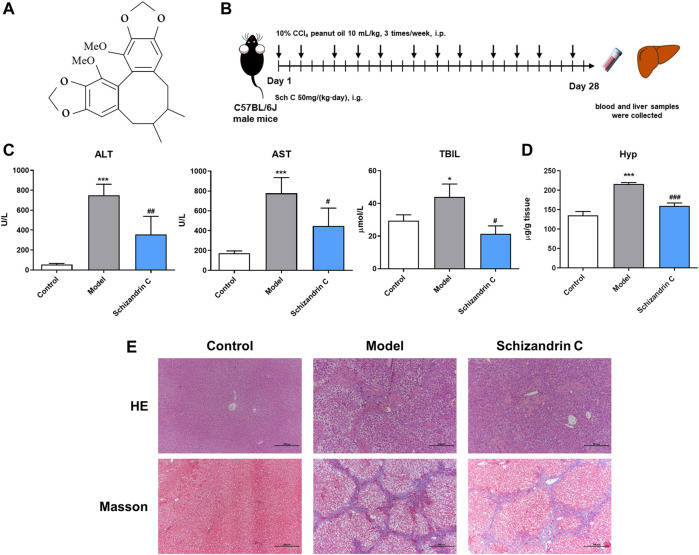
Schizandrin C attenuates liver fibrosis induced by CCl_4_ in C57BL/6J mice. **(A)** The chemical structure of Schizandrin C. **(B)** Schematic presentation of the animal experiment. **(C)** The activities of ALT, AST, and TBIL in serum, n = 3–5. **(D)** Hydroxyproline contents in the liver, n = 5. **(E)** Representative pictures of H&E and Masson staining in the liver. Data are expressed as mean ± S.D. ^*^ *p* < 0.05 and ^***^ *p* < 0.001 versus the control group; ^#^ *p* < 0.05, ^##^ *p* < 0.01, and ^###^ *p* < 0.001 versus model group. Sch C, Schizandrin C.

Male C57BL/6J mice, which were 6–8 week-old and 18–22 g, were purchased from Vital River Laboratory Animal Technology Co., Ltd. (Beijing, China). The mice were raised under controlled conditions with 21°C ± 2°C, 50% ± 10% humidity, and 12 h light–dark cycles. All experimental operating procedures followed the guidelines of the Animal Ethics Committee of Shanghai Ninth People’s Hospital, Shanghai JiaoTong University School of Medicine (Approval ID: SH9H-2019-A574-1). Then, 10 mL/kg 10% CCl_4_ peanut oil was intraperitoneally injected into the mice 3 times/week for 4 weeks to induce liver fibrosis. At the same time, Schizandrin C (Chishao Biotech Co., Ltd., Shanghai, China) at 50 mg/kg was orally administered once a day to the liver fibrosis model mice. The control mice were administered equivalent volumes of peanut oil and 0.5% carboxymethylcellulose solution (CMC-Na). Then, 4 weeks later, the mice were euthanized to obtain blood samples and liver tissues. The structure of Schizandrin C (C_22_H_24_O_6_, CAS No: 61301-33-5- purity ≥98%) is presented in Figure 1A.

### 2.2 Biochemical evaluation

Blood was centrifugated at 1,000 *g* for 15 min to obtain serum. The activities of alanine aminotransferase (ALT), aspartate aminotransferase (AST), and total bilirubin (TBIL) were analyzed using an automatic biochemical analyzer (Chemray 240, Kayto, Shenzhen, China) as per the manufacturer’s instructions.

### 2.3 Histologic evaluation

After acquiring the liver tissues, the liver’s left lobes were separated, fixed in formaldehyde, embedded with paraffin, and sectioned. Then paraffin sections were stained with hematoxylin and eosin (H&E) or Masson’s trichrome staining in accordance with the standard protocol. The staining results were taken using a fluorescence microscope (Nikon Eclipse Ni, Japan).

### 2.4 RNA extraction and quantitative real-time PCR analysis

Liver samples were crushed using a TL2020 grinding instrument (DHS Life Science & Technology, Beijing, China). Total RNA was isolated by Trizol reagent (Invitrogen, United States) as per the standard protocol. Quantitative real-time PCR analysis was performed using One-Step TB GreenTM PrimeScript PLUS RT-PCR Kit (TaKaRa, Japan) in a LightCycler 480II instrument (Hoffman-La Roche Ltd., Basel, Switzerland). The values were analyzed by using the 2^−ΔΔCT^ method. The primer sequences are presented in Supplementary Table S1.

### 2.5 Immunofluorescence staining

After liver tissue sections were deparaffinized, the paraffin sections were blocked with bovine serum albumin (BSA) and incubated with antibodies against alpha-smooth muscle actin (α-SMA) (1:500, Servicebio, Cat# GB111364), type Ι collagen (collagen I) (1:1,000, Servicebio, Cat# GB11022-3), phospho (p)-nuclear factor kappa-B (NF-κB) p65 (1:200, Servicebio, Cat# GB13142-1), p-p38 MAP kinase (p38 MAPK) (1:200, Servicebio, Cat# GB13006-1), and p-extracellular signal-regulated protein kinase (ERK) (1:300, Servicebio, Cat# GB13004-1) overnight at 4°C. Then, the paraffin sections were treated with fluorescence-conjugated secondary antibodies (1:400, Servicebio, Cat# GB25303) and counterstained with 40, 6-diamidino-2-phenylindole (DAPI) (Servicebio, Cat# GB1012). The staining results were acquired using a Pannoramic MIDI scanner (3DHISTECH, Hungary) and viewed by using CaseViewer 2.4 (3DHISTECH, Hungary).

### 2.6 Western blot analysis

Liver total protein was isolated by RIPA buffer and quantified by a BCA protein assay kit (Thermo Fisher Scientific, United States) according to the manufacturer’s instructions. Proteins were separated through an SDS-PAGE gel and transferred onto polyvinylidene difluoride (PVDF) membranes and then blocked in 5% skimmed milk. Afterward, the membranes were incubated with the primary antibody against IκB Kinase-β (IKKβ) (Cell Signaling Technology, Cat# 8943), NF-κB p65 (Cell Signaling Technology, Cat# 8242), p-NF-κB p65 (Cell Signaling Technology, Cat# 3033), ERK (Cell Signaling Technology, Cat# 4695), p-ERK (Cell Signaling Technology, Cat# 4370), p38 (Cell Signaling Technology, Cat# 8690), p-p38 (Cell Signaling Technology, Cat# 4511), and β-tubulin (Servicebio, Cat# GB122667) at 4°C overnight and then with the corresponding anti-rabbit secondary antibody (Cell Signaling Technology, Cat# 7074) or anti-mouse secondary antibody (Cell Signaling Technology, Cat# 7076) at room temperature for 1 h. Finally, the bands were determined using a chemiluminescence apparatus (Tanon 5200, China) and quantified using the Image J software (National Institute of Mental Health, United States).

### 2.7 Lipidomics analysis

First, 100 mg of liver tissues were treated with 0.75 mL of methanol in a glass tube with a Teflon-lined cap. After vortexing, 2.5 mL of methyl tert-butyl ether (MTBE) was added and incubated in a shaker for 1 h at room temperature. Afterward, the samples were added to 0.625 mL of MS-grade water, incubated for 10 min at room temperature, and centrifuged at 1,000 g for 10 min. The upper phase was gathered, and the lower phase was re-extracted using 1 mL of MTBE/methanol/water (10:3:2.5, v/v/v) for the organic phase. Combined organic phases were dried under nitrogen and re-dissolved in 100 μL of isopropanol for ultra-high-performance liquid chromatography–tandem mass spectrometry (UHPLC-MS/MS) analysis by using a Vanquish UHPLC system (Thermo Fisher, Germany) with an Orbitrap Q ExactiveTM HF mass spectrometer (Thermo Fisher, Germany) in Novogene Co., Ltd. (Beijing, China).

A Thermo Accucore C30 column (150 × 2.1 mm, 2.6 μm) was used, and the samples were injected with a linear gradient flow rate of 0.35 mL/min for 20 min. The temperature of the column was set at 40°C. Acetonitrile/water (6/4) coupled with 10 mM ammonium acetate and 0.1% formic acid was used as mobile phase buffer A, and acetonitrile/isopropanol (1/9) coupled with 10 mM ammonium acetate and 0.1% formic acid was used as buffer B. The solvent gradient was initially 30% B for 2 min and then 43% B for 5 min, 55% B for 5.1 min, 70% B for 11 min, 99% B for 16 min, and 30% B for 18.1 min.

Raw data were processed by using Compound Discoverer 3.01 (CD3.1, Thermo Fisher) for peak alignment, peak picking, and quantitation. Then, peak intensities were normalized to the total spectral intensity and used to calculate the molecular formula on account of additive ions, molecular ion peaks, and fragment ions. The data were matched for accurate qualitative and relative quantitative results using LIPID MAPS, LipidBlast, and HMDB databases. If data were not distributed normally, area normalization was attempted used for normal transformation. Principal components analysis (PCA) and orthogonal partial least squares discriminant analysis (OPLS-DA) were operated using MetaboAnalyst 5.0 (www.metaboanalyst.ca). Potential differential metabolites were picked with variable importance in projection (VIP) > 1 and *p* < 0.05, or fold change (FC) ≥ 2, or FC ≤ 0.5 (Supplementary Table S2).

### 2.8 Hepatic hydroxyproline determination

The hepatic hydroxyproline (HYP) content was measured using a hydroxyproline content detection kit (Fushen Biotech, China).

### 2.9 Cell culture

Hepatic stellate cell lines LX-2 (CVCL_5792) and HSC-T6 (CVCL_0315) were acquired from FuHeng Cell Center (Shanghai, China). Both cell lines were cultured in high glucose Dulbecco’s modified Eagle’s medium (DMEM) with 10% FBS and maintained in an incubator with 5% CO_2_ at 37°C. Cell Counting Kit-8 (CCK-8, Fushen Biotech, China) was used to screen suitable concentrations of Schizandrin C as per the manufacturer’s instructions, and TGF-β1 at 20 ng/mL was used to activate HSCs.

### 2.10 Statistical analysis

The values are shown as mean ± SD. Multiple comparisons were conducted with one-way ANOVA, followed by the Student–Newman–Keuls *post hoc* test. All data were analyzed by GraphPad Prism 8.0 (GraphPad Software Incorporated, United State).

## 3 Results

### 3.1 Schizandrin C attenuates liver fibrosis induced by CCl_4_ in C57BL/6J mice

To evaluate the effect of Schizandrin C on liver fibrosis, male C57BL/6J mice were raised, and CCl_4_ was used to establish a liver fibrosis model (Figure 1B). As shown in Figure 1C, serum ALT, AST, and TBIL were significantly decreased upon treatment with Schizandrin C in CCl_4_ mice. In addition, the hepatic hydroxyproline content was raised notably in model group and reduced by treatment with Schizandrin C (Figure 1D). H&E and Masson staining results displayed that Schizandrin C markedly attenuated the degree of liver necrosis and collagen deposition stimulated by CCl_4_ (Figure 1E). Overall, Schizandrin C can attenuate liver fibrosis induced by CCl_4_ in C57BL/6J mice.

### 3.2 Schizandrin C attenuates HSC activation in CCl_4_ mice and TGF-β1-treated HSCs

**FIGURE 2:**
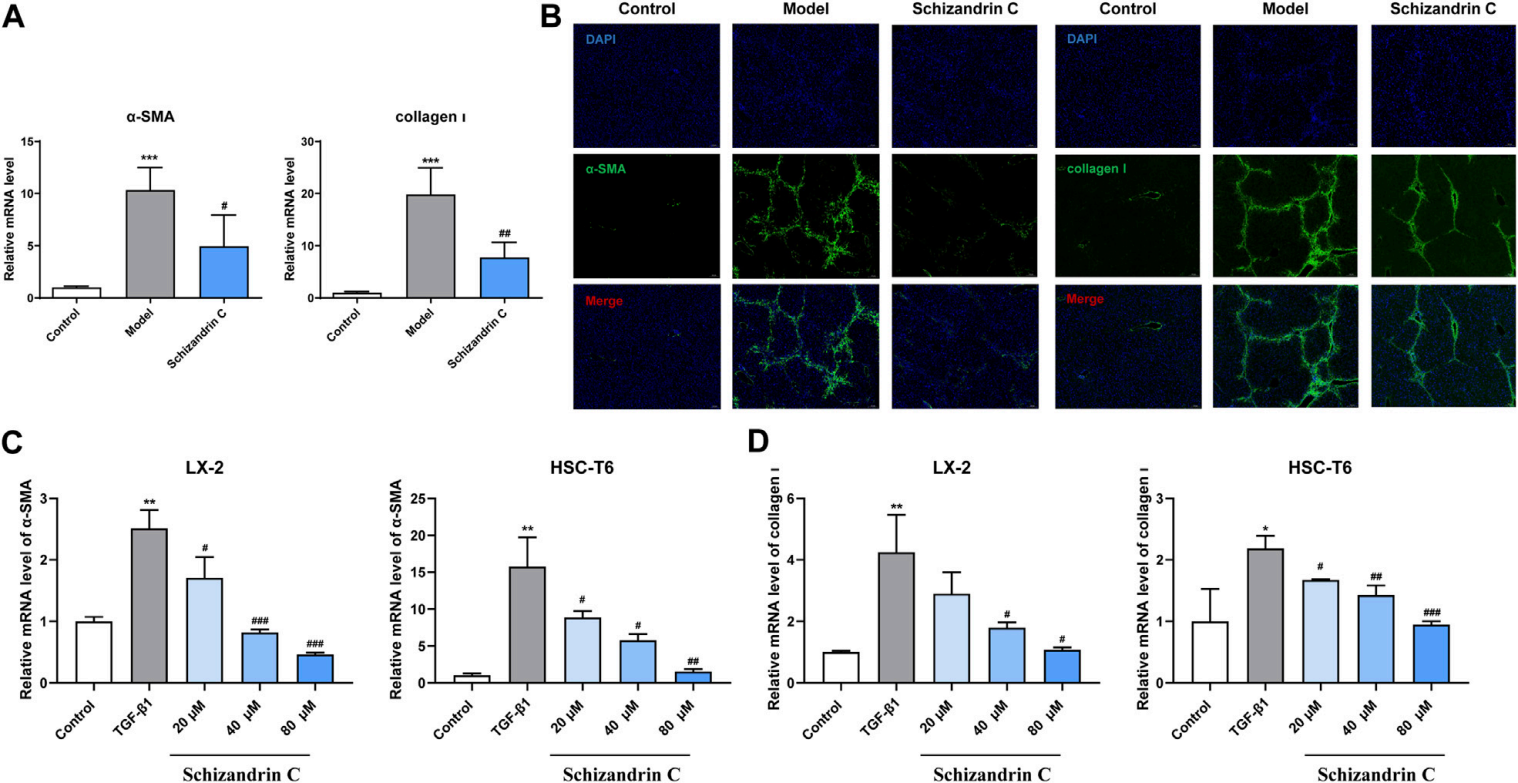
Schizandrin C attenuates HSC activation in CCl_4_ mice and TGF-β1-treated HSCs. **(A)** The mRNA levels of α-SMA and collagen Ⅰ in the liver, n = 5. **(B)** Representative results of immunofluorescence staining for α-SMA and collagen Ⅰ in the liver. **(C)** The mRNA level of α-SMA in LX-2 and HSC-T6 cells treated with Schizandrin C, n = 3. **(D)** The mRNA level of collagen Ⅰ in LX-2 and HSC-T6 cells treated with Schizandrin C, n = 3. Data are expressed as mean ± S.D. ^*^ *p* < 0.05, ^**^ *p* < 0.01, and ^***^ *p* < 0.001 versus the control group; ^#^ *p* < 0.05, ^##^ *p* < 0.01, and ^###^ *p* < 0.001 versus model or TGF-β1 groups.

As shown in Figure 2A, the mRNA levels of α-SMA and collagen Ⅰ activated by CCl_4_ were significantly decreased upon treatment of Schizandrin C. Immunofluorescence staining results also showed that the high protein levels of α-SMA and collagen Ⅰ in CCl_4_ mice liver were downregulated by treatment with Schizandrin C (Figure 2B). To further identify the effect of Schizandrin C on HSC activation, different concentrations of Schizandrin C were treated in LX-2 and HSC-T6 cells to assess the toxicity of Schizandrin C on cells (Supplementary Figures S1A, B). Then, LX-2 and HSC-T6 cells were activated by TGF-β1, and Schizandrin C at 20, 40, and 80 μM was used to treat the cells. As shown in Figures 2C, D, Schizandrin C at 20, 40, and 80 μM depressed the mRNA levels of α-SMA and collagen Ι raised by TGF-β1 both in LX-2 and HSC-T6 cells. Thus, Schizandrin C can attenuate HSC activation *in vivo* and *in vitro*.

### 3.3 Schizandrin C affects the liver lipid profile in CCl_4_ mice

**FIGURE 3:**
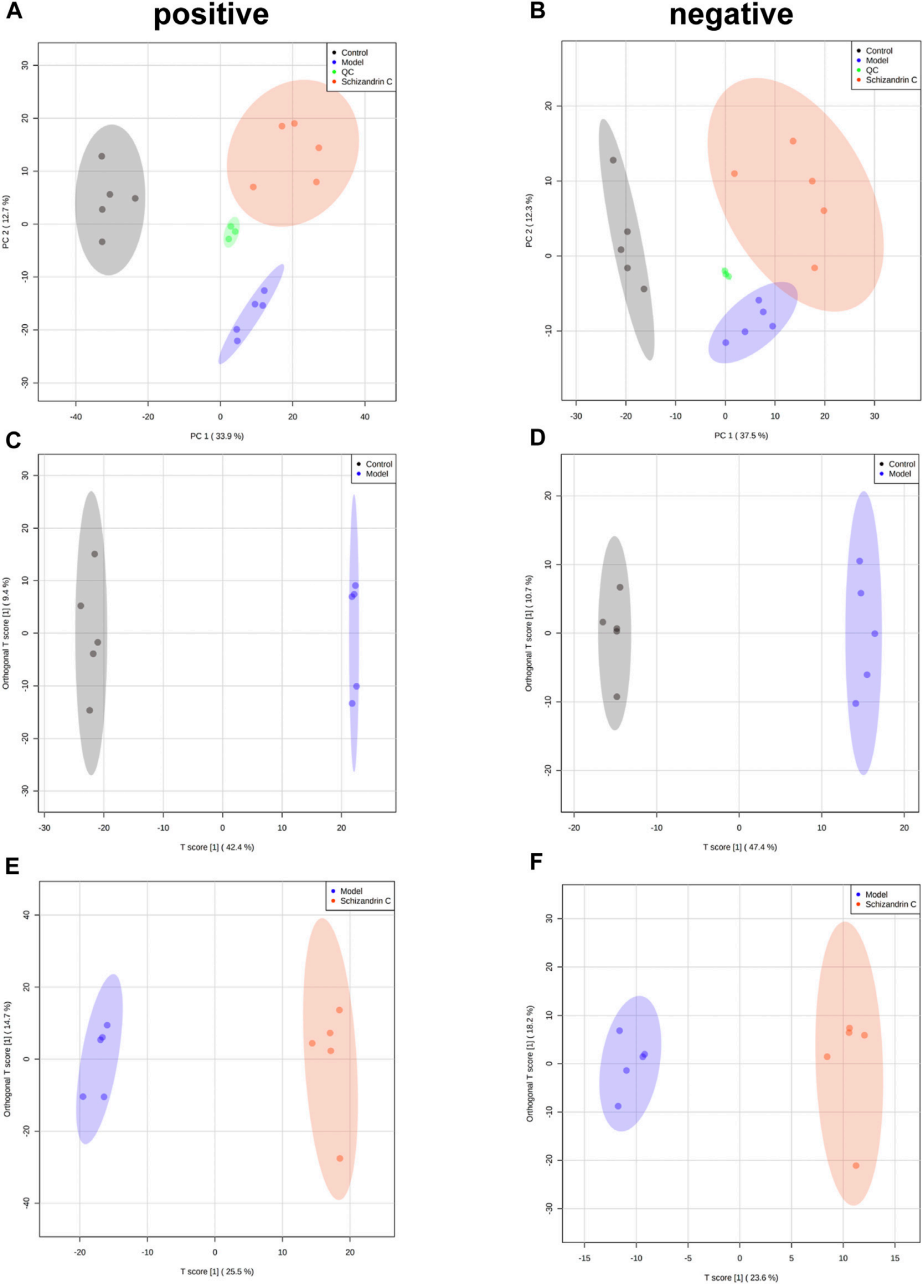
Schizandrin C affects the liver lipid profile in CCl_4_ mice. PCA score plots of liver samples in positive **(A)** and negative **(B)** ion modes. OPLS-DA score plots of liver samples between model and control groups in positive **(C)** and negative **(D)** ion modes. OPLS-DA score plots of liver samples between Schizandrin C and model groups in positive **(E)** and negative **(F)** ion modes.

To further explore the effect of Schizandrin C on lipid metabolism, lipidomics was performed, and PCA was used to display the clustering separation among the original data of the three groups. As shown in Figures 3A, B, the QC samples clustered closely with no drift. In addition, samples of the CCl_4_ model group were markedly separated from those of the control and Schizandrin C groups, suggesting that CCl_4_ stimulated the liver lipid profile change and Schizandrin C can affect lipid metabolism in CCl_4_ mice. In addition, OPLS-DA was performed to determine the lipid variations between the model and control groups, and Schizandrin C and model groups in positive and negative ion modes. After 1,000 permutations, the intercept value of R2Y and Q2 was 0.999 and 0.958, 0.997 and 0.954, respectively, in positive and negative ion modes between the model and control groups (Supplementary Figures S2A, B). In addition, the intercept value of R2Y and Q2 was 0.994 and 0.821, 0.988 and 0.747, respectively, in positive and negative ion modes between the Schizandrin C and model groups (Supplementary Figures S2C, D). The values of R2Y and Q2 indicated that OPLS-DA models conformed to the true situation of sample data with a low risk of overfitting and reliability. As shown in Figures 3C–F, OPLS-DA score plots suggested that the model and control groups, and Schizandrin C and model groups separated notably in positive and negative ion modes. These results displayed that Schizandrin C can affect the liver lipid profile in CCl_4_ mice.

### 3.4 Schizandrin C regulates lipid metabolism in liver fibrosis

**FIGURE 4:**
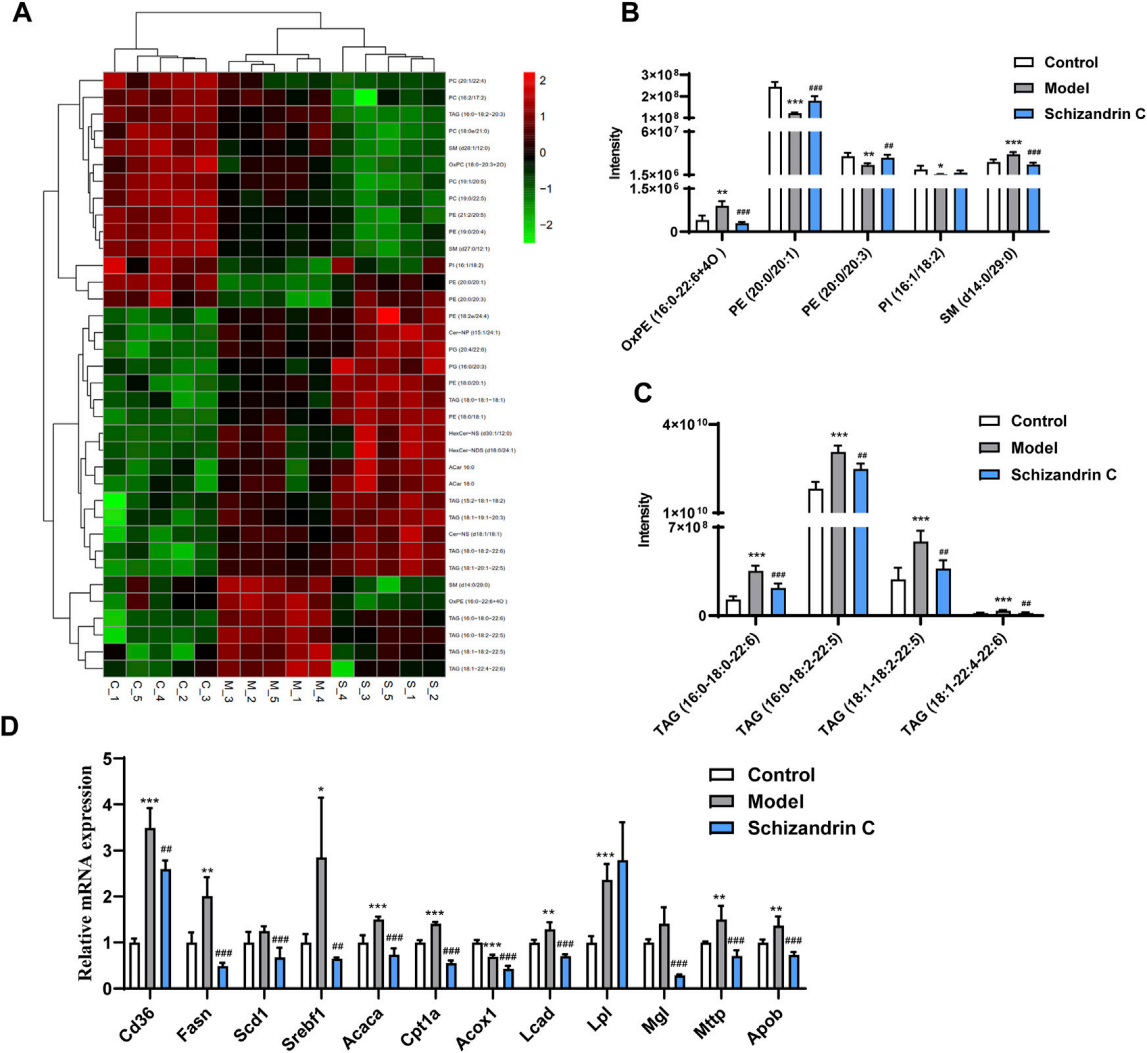
Schizandrin C regulates lipid metabolism in liver fibrosis. **(A)** Heat map of potential biomarkers from model versus control groups and Schizandrin C versus model groups of liver samples. **(B, C)** Responses of the potential markers in the three groups, n = 5. **(D)** The mRNA levels of enzymes or transporters related to lipid metabolism in liver of the three groups, n = 5. Data are expressed as mean ± S.D. ^*^ *p* < 0.05, ^**^ *p* < 0.01, and ^***^ *p* < 0.001 versus the control group; ^##^ *p* < 0.01 and ^###^ *p* < 0.001 versus the model group.

Then, an S-plot was further employed to identify altered lipids that significantly contributed to the differences between the model and normal groups or Schizandrin C and model groups (Supplementary Figures S3A–D). Varied lipids with VIP>1, *p* < 0.05, FC ≥ 2, or FC ≤ 0.5 were further selected for analysis. Those lipid species which were differential simultaneously in model vs. control groups and Schizandrin C vs. model groups were considered pivotal potential biomarkers for the Schizandrin C treatment of liver fibrosis (Figure 4A). A total of 36 lipid species, including ten triacylglycerols (TAGs), eight phosphatidylethanolamines (PEs), six phosphatidylcholines (PCs), three sphingomyelins (SMs), two acylcarnitines (ACars), two ceramides (Cers), two glucosylceramides (GlcCers), two phosphatidylglycerols (PGs), and one phosphatidylinositol (PI) were differentially regulated in the liver. What is more, PE (20:0/20:1), PE (20:0/20:3) and PI (16:1/18:2) levels were markedly decreased in model group, but notably increased by Schizandrin C treatment (Figure 4B). In addition, OxPE (16:0–22:6+4O), SM (d14:0/29:0), TAG (16:0-18:0-22:6), TAG (16:0-18:2–22:5), TAG (18:1-18:2-22:5), and TAG (18:1-22:4-22:6) levels were significantly increased in the model group but reversed by Schizandrin C supplementation (Figures 4B, C). Finally, the effect of Schizandrin C on lipid metabolism-associated gene expressions was determined. It is obvious that the mRNA levels of CD36 molecule (*Cd36*), fatty acid synthase (*Fasn*), sterol regulatory element-binding transcription factor 1 (*Srebf1*), acetyl-coenzyme A carboxylase alpha (*Acaca*), carnitine palmitoyltransferase 1a (*Cpt1a*), long-chain acyl-CoA dehydrogenase (*Lcad*)*,* lipoprotein lipase (*Lpl*), microsomal triglyceride transfer protein (*Mttp*), and apolipoprotein B (*Apob*) were markedly increased, and treatment with Schizandrin C notably decreased *Cd36*, *Fasn*, stearoyl-coenzyme A desaturase 1 (*Scd1*), *Srebf1*, *Acaca*, *Cpt1a*, acyl-coenzyme A oxidase 1 (*Acox1*), *Lcad*, monoglyceride lipase (*Mgl*), *Mttp*, and *Apob* (Figure 4D). Together, these results indicated that Schizandrin C can reverse lipid disorder induced by CCl_4_.

### 3.5 Schizandrin C improves inflammation and inhibits the NF-κB signaling pathway

**FIGURE 5:**
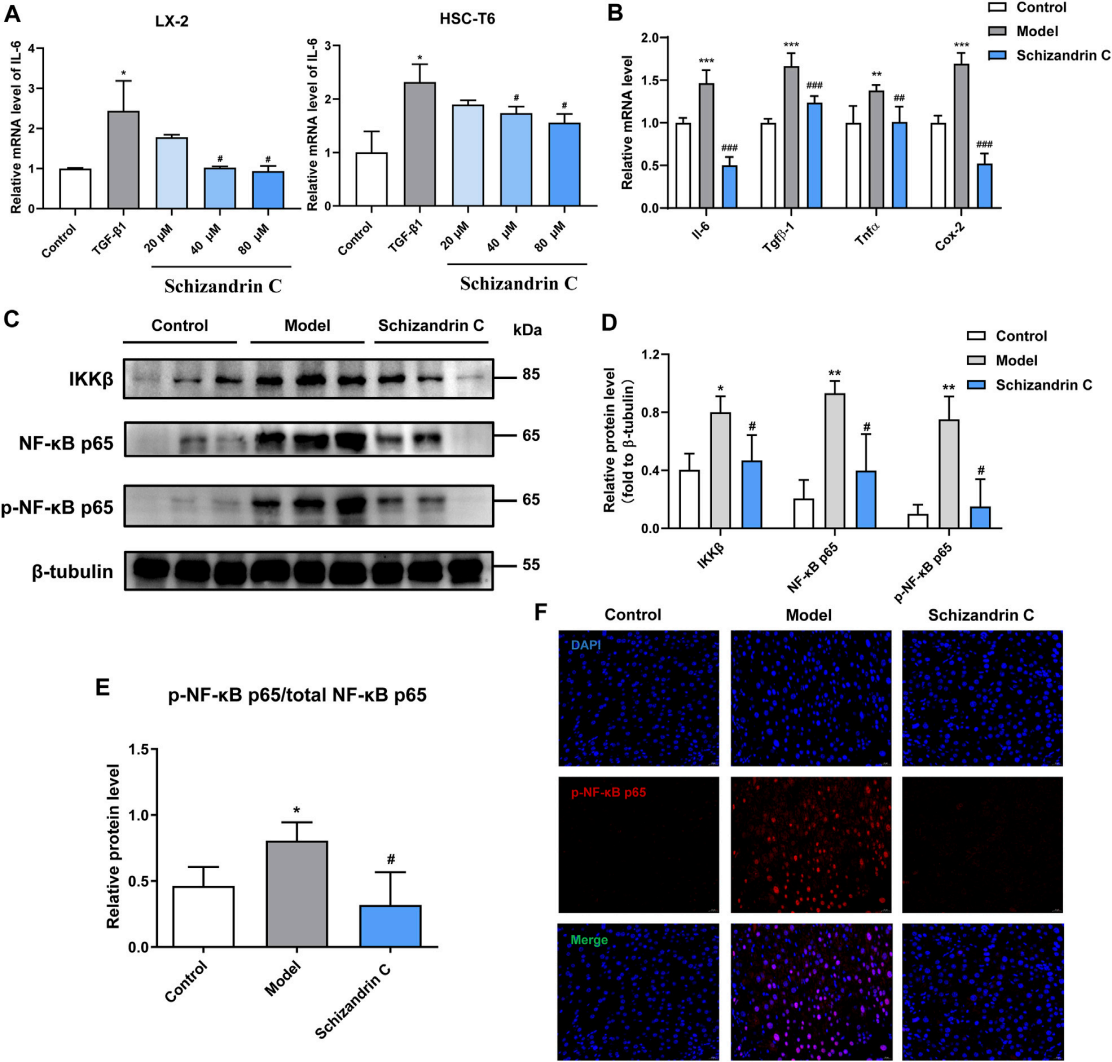
Schizandrin C improves inflammation and inhibits the NF-κB signaling pathway. **(A)** The mRNA level of IL-6 in LX-2 and HSC-T6 cells treated with Schizandrin C, n = 3. **(B)** The mRNA levels of the inflammatory factors in liver of the three groups, n = 5. **(C)** The images of NF-κB signaling-associated proteins detected by Western blotting. **(D, E)** Densitometric analysis for NF-κB signaling-associated protein levels, n = 3. **(F)** Representative results of immunofluorescence staining for p-NK-κB p65. Data are expressed as mean ± S.D. ^*^ *p* < 0.05, ^**^ *p* < 0.01, and ^***^ *p* < 0.001 versus the control group; ^#^ *p* < 0.05, ^##^ *p* < 0.01, and ^###^ *p* < 0.001 versus model or TGF-β1 groups.

As shown in Figure 5A, the mRNA level of interleukin 6 (IL-6) was stimulated by TGF-β1, and treatment with Schizandrin C concentration-dependently suppressed it in both LX-2 and HSC-T6 cells. Furthermore, Schizandrin C notably reduced the mRNA levels of inflammation factors induced by CCl_4_ in the liver, including *Il-6,* transforming growth factor beta 1 (*Tgfβ-1*)*,* tumor necrosis factor (*Tnfα*), and cyclooxygenase-2 (*Cox-2*) (Figure 5B). Then, inflammation-related NF-κB signaling pathway was further detected. It is notable that Schizandrin C decreased the protein levels of IKKβ, NF-κB p65, and p-NF-κB p65, which were activated by CCl_4_ (Figures 5C, D). In addition, the phosphorylation level of NF-κB p65 was inhibited by treatment with Schizandrin C (Figure 5E). Immunofluorescence staining results also displayed that high p-NF-κB p65 expression in the nucleus was attenuated by treatment with Schizandrin C in CCl_4_ liver (Figure 5F). These results suggested that Schizandrin C can improve liver inflammation and inhibit the NF-κB signaling pathway in CCl_4_ mice.

### 3.6 Schizandrin C inhibits the p38/ERK MAPK signaling pathway

**FIGURE 6:**
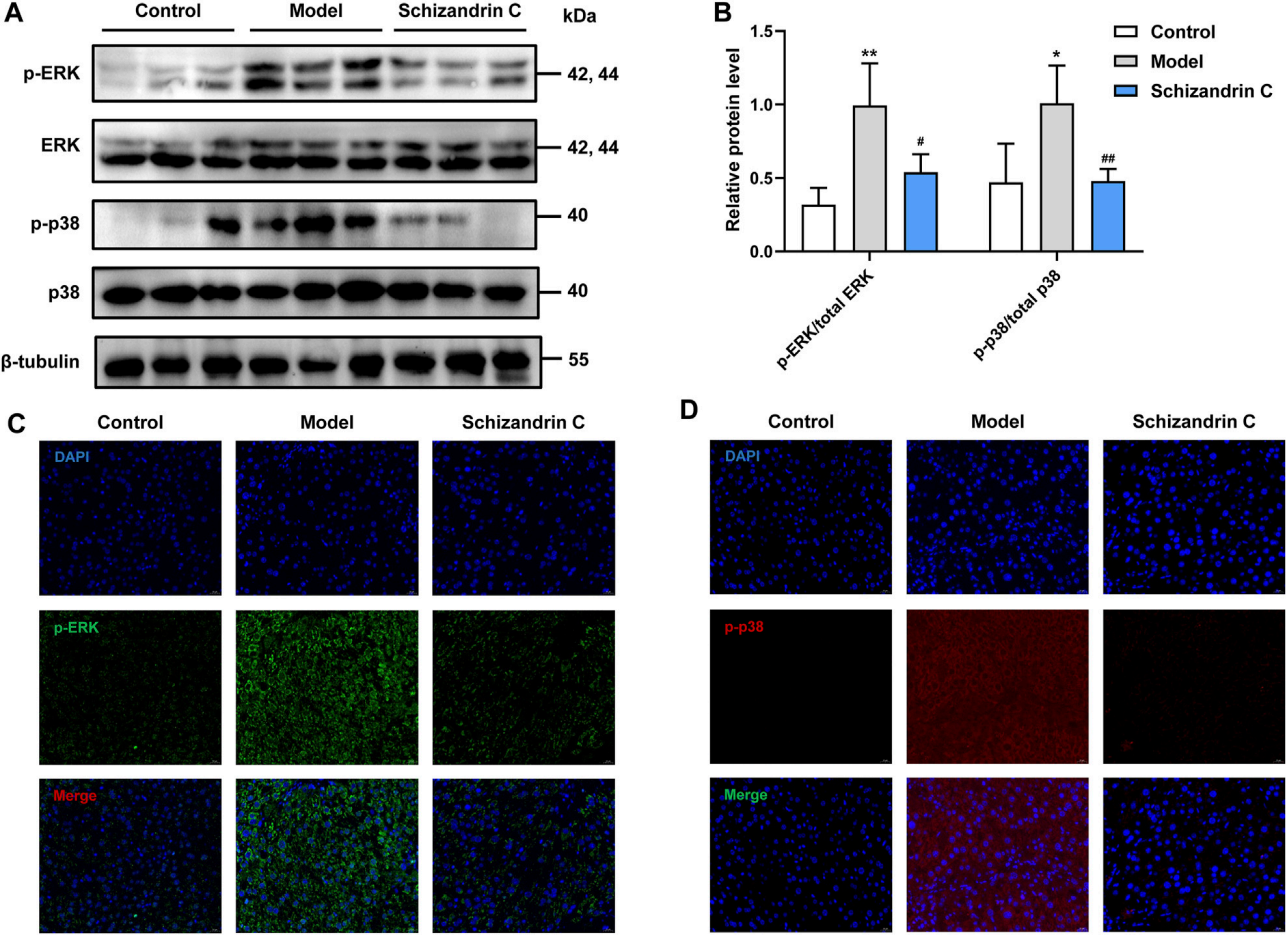
Schizandrin C inhibits the p38/ERK MAPK signaling pathway. **(A)** The images of MAPK signaling-associated proteins detected by Western blotting. **(B)** Densitometric analysis for MAPK signaling-associated proteins, n = 5. Representative results of immunofluorescence staining for p-ERK **(C)** and p-p38 **(D)**. Data are expressed as mean ± S.D. ^*^ *p* < 0.05 and ^**^ *p* < 0.01 versus the control group; ^#^ *p* < 0.05 and ^##^ *p* < 0.01 versus the model group.

As the mitogen-activated protein kinase (MAPK) signaling pathway is closely associated with lipid metabolism, inflammation, and liver fibrosis, relative protein levels were then detected. Western blot analysis showed that the total protein levels of p38 and ERK were basically unchanged, but phosphorylation levels of ERK and p38 were higher in CCl_4_ liver, and treatment with Schizandrin C reduced the protein levels of p-ERK and p-p38 (Figures 6A,B). The same trends can be seen in immunofluorescence staining images (Figures 6C, D). Taken together, Schizandrin C can inhibit the p38/ERK MAPK signaling pathway in CCl_4_ mice.

## 4 Discussion

Evidence has showed that liver fibrosis can cause liver architecture and functional impairment, resulting in cirrhosis and organ failure (Pinzani et al., 2005; Khurana et al., 2021). If the cause of injury is removed, fibrosis can be reversed and solved in different organs, especially in the liver, with a high regenerative capacity (Caligiuri et al., 2021). In this study, we found that Schizandrin C, a lignan from Schisandra chinensis, exerts anti-hepatic fibrosis function, and the mechanisms were associated with NF-κB signaling- and MAPK signaling-mediated inflammation and lipid homeostasis.

Almost all chronic liver injuries induced by different etiologies may contribute to liver fibrosis (Lai and Afdhal, 2019; Liu et al., 2023). When a liver develops fibrosis, a large number of extracellular matrices, such as collagen Ι, will deposit excessively since quiescent HSCs are activated into contractile myofibroblast-like cells (Brempelis and Crispe, 2016; Xu et al., 2016; Lyu et al., 2023; Zhang et al., 2023). Meanwhile, these activated HSCs express abundant α-SMA, which is thought to be a marker for HSC activation and liver fibrosis (Nouchi et al., 1991; Friedman, 2008). Thus, inhibition of HSC activation is considered a vital event that contributes to control liver fibrosis (Zhang et al., 2022; Lyu et al., 2023). In this study, CCl_4_ mice showed a higher level of liver function-related markers in serum, raised hepatic hydroxyproline content, impaired structure, and deposited collagen in the liver, and treatment with Schizandrin C decreased the disturbed serum ALT, AST, and TBIL, reduced hepatic hydroxyproline content, and weakened liver damage and collagen accumulation. Furthermore, the expressions of α-SMA and collagen Ι activated by CCl_4_ were reduced by Schizandrin C *in vivo*. The *in vitro* experiment also indicated that HSC activation was suppressed by Schizandrin C. All these results suggested that Schizandrin C exerts a significant anti-liver fibrosis effect.

The liver controls the central processes of lipid homeostasis, and disturbed lipid metabolism can contribute to the occurrence and progression of liver fibrosis (Moustafa et al., 2012; He et al., 2022). Here, lipidomics was performed, and the results displayed that lipid profiles were varied between the CCl_4_ model and normal liver samples, and treatment with Schizandrin C can regulate lipid species in CCl_4_ liver. Previous studies have displayed that Schizandrin C may inhibit CCl_4_-activated lipid peroxidation and the combination of CCl_4_ metabolites and lipids (Liu and Lesca, 1982; Lu and Liu, 1991). However, only OxPE (16:0-22:6+4O) was found to be a remarkable specie that may be associated with the inhibitory effect of Schizandrin C on lipid peroxidation. It was reported that total PC and PE in fibrotic livers of CCl_4_ rats decreased markedly, and most PCs and PEs decreased to a great extent (Ahn et al., 2008). Here, we also found that most phospholipids were decreased in fibrotic livers, but PE (20:0/20:1), PE (20:0/20:3), and PI (16:1/18:2) were upregulated by Schizandrin C, suggesting that these three species are notable markers that associated with Schizandrin C improving the liver structure. Sphingolipids, ubiquitous components in eukaryotic cell membranes, are structural lipids and signaling molecules to regulate pivotal cellular functions, including immune responses in health and metabolic disorders (Rodriguez-Cuenca et al., 2017). In this study, SM (d14:0/29:0), which was upregulated in the fibrotic liver but decreased by Schizandrin C treatment, may be a notable sphingolipid regulated by Schizandrin C treatment. Additionally, it is worth noting that TAG (16:0-18:0-22:6), TAG (16:0-18:2-22:5), TAG (18:1-18:2-22:5), and TAG (18:1-22:4-22:6) were increased in the fibrotic liver but reduced by Schizandrin C treatment. Various studies have shown that TAG accumulation is a key factor triggering lipid spectrum disorders and metabolic syndromes (Liu et al., 2022). Here, four TAG species were identified as markers for Schizandrin C therapy on liver fibrosis. Furthermore, the genes associated with lipid homeostasis were all altered in fibrotic liver, including *Cd36* associated with lipid uptake, *Fasn*, *Scd1*, *Srebf1*, and *Acaca* associated with lipid synthesis, *Cpt1a*, *Acox1*, *Lcad*, and *Mgl* associated with lipid metabolism, and *Mttp* and *Apob* associated with TAG transfer. However, treatment with Schizandrin C can reverse most mRNA levels of these enzymes or transporters, further suggesting that Schizandrin C can regulate lipid metabolism in CCl_4_ fibrotic liver.

Recent research studies have reported that TAG metabolism is closely associated with inflammation (Castoldi et al., 2020; Puig et al., 2020; Macpherson et al., 2023). Though inflammation is beneficial for the regeneration of impaired liver tissue, persistent chronic inflammation can cause a permanent healing state, subsequently initiating liver fibrosis (de Souza Basso et al., 2021). Especially, HSCs may respond to immunological triggers and acquire a pro-inflammatory profile by expressing more inflammatory genes, including IL-6, during liver fibrosis (Najar et al., 2017). It is well known that NF-κB is an essential transcriptional regulator of inflammatory response in the liver (Luedde and Schwabe, 2011). NF-κB is located in the cytoplasm and combines with inhibitory proteins and some precursor proteins normally (Schmitz et al., 2004). When the stimulus activates IKK, NF-κB is released from its inhibitory proteins and translocates to the nucleus, followed by activating gene transcription (Karin and Ben-Neriah, 2000). Evidence has showed that NF-κB can modulate liver fibrosis predominantly via the regulation of hepatocyte injury, fibrogenic responses in HSCs, and inflammatory signals induced by macrophages and other inflammatory cells (Luedde and Schwabe, 2011). In addition, phosphorylation and nuclear translocation of the NF-κB p65 subunit are indicators to assess the activation and functional status of the NF-κB pathway (Pradère et al., 2016). Then, it continuously regulates pro-inflammatory factors, such as IL-6 and TNF-α (Romero et al., 2020), which are the main triggers of excessive accumulation of ECM in the fibrotic liver (Afratis et al., 2018). In our study, *IL-6* was increased by TGF-β1 stimulation in both LX-2 and HSC-T6 cells and decreased by treatment with Schizandrin C. More importantly, the protein levels of IKKβ, NF-κB p65, and p-NF-κB p65, especially the phosphorylation level of NF-κB p65, were elevated, and inflammatory factors, such as *Il-6*, *Tgfβ-1*, *Tnfα*, and *Cox-2,* were raised in CCl_4_ fibrotic liver, but treatment with Schizandrin C can inhibit them, indicating that Schizandrin C can attenuate inflammation and the NF-κB signaling pathway.

MAPK pathways, such as p38 MAPK and ERK, are significant for cell growth and differentiation, and closely associated with liver fibrosis (Li et al., 2016). Previous evidence has demonstrated that the MAPK pathway is related to NF-κB activation stimulated by lipopolysaccharide (LPS) (Guha and Mackman, 2001; Wu et al., 2023). It is worth noting that both MAPK and NF-κB are regarded as classical and key anti-inflammatory pathways, and MAPK can activate NF-κB to regulate inflammatory processes (Kim et al., 2019; Ren et al., 2020; Tang et al., 2021). In addition, some lipids or lipid-soluble extracts can inhibit inflammatory response via NF-κB and MAPK in LPS-activated macrophages (Si et al., 2016; Choi et al., 2022). Furthermore, growing evidence has displayed that blunting MAPK activities is an effective strategy to depress liver fibrosis via augmenting NF-κB inactivation and attenuating lipid accumulation (Abdelhamid et al., 2021; Wang et al., 2021). Here, we found that Schizandrin C reduced the phosphorylation of p38 MAPK and ERK, suggesting that Schizandrin C attenuates liver fibrosis, regulates lipid metabolism, inhibits inflammation response and NF-κB activity was associated with the p38/ERK MAPK signaling pathway.

In conclusion, Schizandrin C can attenuate liver fibrosis induced by CCl_4_, which is possibly via the regulation of lipid metabolism and inflammation mediated by NF-κB and p38/ERK MAPK signaling pathways. These results support Schizandrin C as a potential therapeutic drug for liver fibrosis.

## Data Availability

The original contributions presented in the study are included in the article/Supplementary Material; further inquiries can be directed to the corresponding author.
