# Supplementary material for: Schizandrin C regulates lipid metabolism and inflammation in liver fibrosis by NF-κB and p38/ERK MAPK signaling pathways
Source: Front Pharmacol. 2023 May 23;14:1092151. doi: 10.3389/fphar.2023.1092151 (PMC10242051; doi:10.3389/fphar.2023.1092151)
Supplement: Supplementary file 1 [file DataSheet1.pdf]

Supplementary Material

1 Supplementary Figures

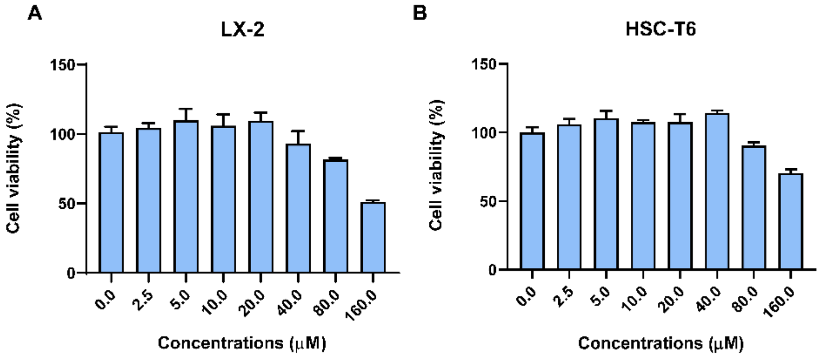

Supplementary Figure 1. The cellular viability of LX-2 (A) and HSC-T6 (B) cells treated with different concentrations of Schizandrin C, n=3. Data are expressed as Mean ± S.D.

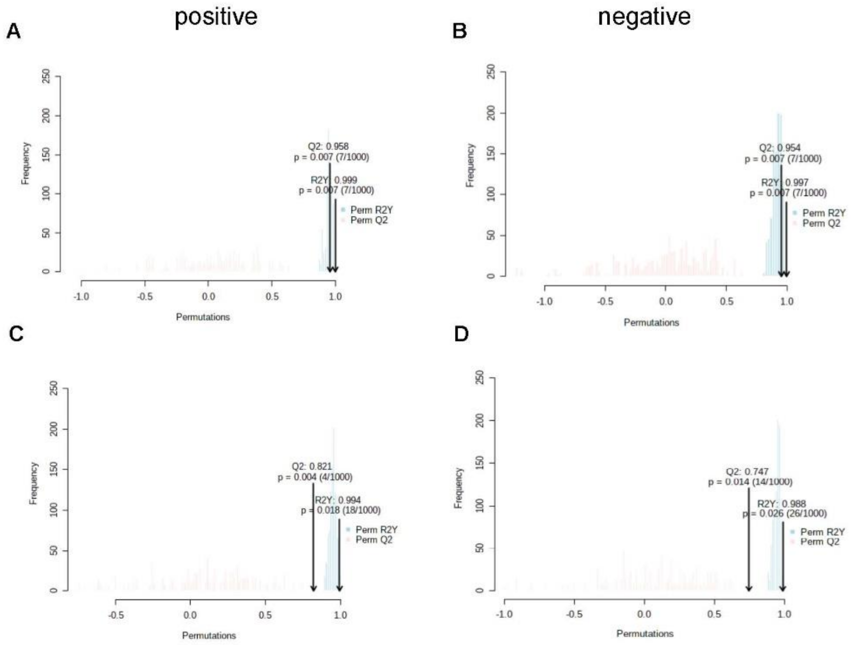

Supplementary Figure 2. Permutation test of OPLS-DA. Permutation plots for model vs. control in positive (A) and negative (B) ion modes. Permutation plots for Schizandrin C vs. model in positive (C) and negative (D) ion modes.

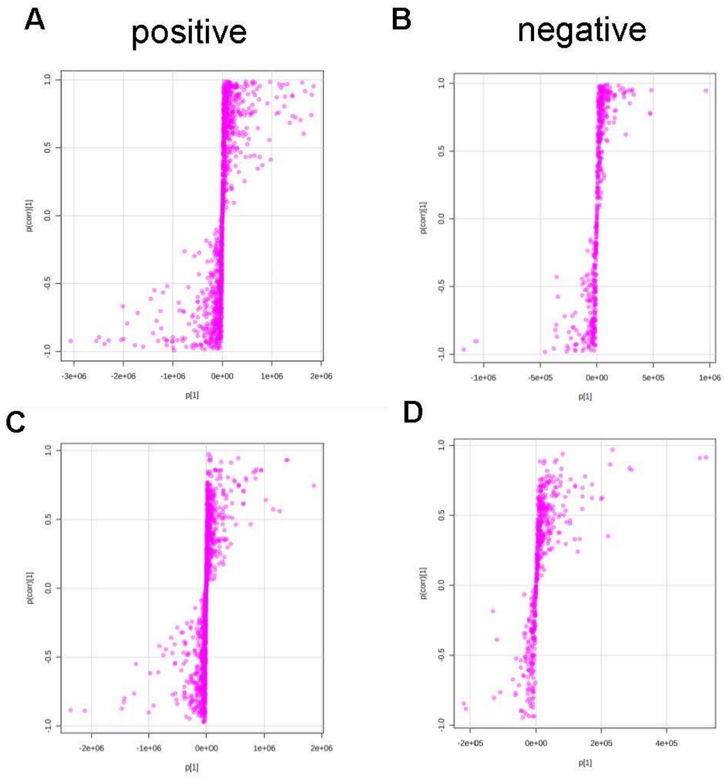

Supplementary Figure 3. S-plot analysis of OPLS-DA. S-plots of relative abundances for model vs. control in positive (A) and negative (B) ion modes. S-plots of relative abundances for Schizandrin C vs. model in positive (C) and negative (D) ion modes.

2 Supplementary Tables

Supplementary Table 1. Sequence of primers for quantitative real-time PCR analysis.

Name	Forward (5'-3')	Reverse (5'-3')
α-SMA	GTCCCAGACATCAGGGAGTAA	TCGGATACTTCAGCGTCAGGA
Col1a1	TAAGGGTCCCCAATGGTGAGA	GGGTCCCTCGACTCCTACAT
Cd36	ATGGGCTGTGATCGGAACTG	GTCTTCCCAATAAGCATGTCTCC
Fasn	GGAGGTGGTGATAGCCGGTAT	TGGGTAATCCATAGAGCCCAG

Scd1	TTCTTGCGATACACTCTGGTGC	CGGGATTGAATGTTCTTGTCGT
Srebf1	TGACCCGGCTATTCCGTGA	CTGGGCTGAGCAATACAGTTC
Acaca	ATGGGCGGAATGGTCTCTTTC	TGGGGACCTTGTCTTCATCAT
Cpt1a	CTCCGCCTGAGCCATGAAG	CACCAGTGATGATGCCATTCT
Acox1	TAACTTCCTCACTCGAAGCCA	AGTTCCATGACCCATCTCTGTC
Lcad	TCTTTTCCTCGGAGCATGACA	GACCTCTCTACTCACTTCTCCAG
Lpl	GGGAGTTTGGCTCCAGAGTTT	TGTGTCTTCAGGGGTCCTTAG
Mgl	ACCATGCTGTGATGCTCTCTG	CAAACGCCTCGGGGATAACC
Mttp	AGCCAGTGGGCATAGAAAATC	GGTCACTTTACAATCCCCAGAG
Apob	AAGCACCTCCGAAAGTACGTG	CTCCAGCTCTACCTTACAGTTGA
Il-6	CTCCCAACAGACCTGTCTATAC	CCATTGCACAACTCTTTTCTCA
Tgfb-1	CTTGCCCTCTACAACCAACA	ACTTGCGACCCACGTAGTAGA
Tnfα	ATGTCTCAGCCTCTTCTCATTC	GCTTGTCACTCGAATTTTGAGA
Cox-2	GGATCATCAACACTGCCTCA	ATGGTGGCTGTCTTGGTAGG
Gapdh	GGTTGTCTCCTGCGACTTCA	TGGTCCAGGGTTTCTTACTCC
α-SMA(rat)	CCAGGGAGTGATGGTTGGA	CCGTTAGCAAGGTCGGATG
Col1a1(rat)	TGTTGGTCCTGCTGGCAAGAATG	GTCACCTTGTTCGCCTGTCTCAC
Il-6 (rat)	ACTTCCAGCCAGTTGCCTTCTTG	TGGTCTGTTGTGGGTGGTATCCTC

Gapdh (rat)	GACATGCCGCCTGGAGAAAC	AGCCCAGGATGCCCTTTAGT
α-SMA (human)	GTGTTGCCCCTGAAGAGCAT	GCTGGGACATTGAAAGTCTCA
COL1A1 (human)	GAGGGCCAAGACGAAGACATC	CAGATCACGTCATCGCACAAC
IL-6 (human)	ACTCACCTCTTCAGAACGAATTG	CCATCTTTGGAAGGTTCAGGTTG
GAPDH (human)	GGAGCGAGATCCCTCCAAAAT	GGCTGTTGTCATACTTCTCATGG

Supplementary Table 2. The information of 36 identified lipids simultaneously in Model vs. Control groups and Schizandrin C vs. Model groups.

**Lipidname**	**m/z**	**Rt (min)**	**Model vs. Control**			**Schizandrin C vs. Model**		
			VIP	*p* value	FC	VIP	*p* value	FC
ACar 16:0	399.33	2.23	1.10	0.02	2.64	1.41	0.02	3.45
ACar 18:0	427.37	3.63	1.08	0.02	2.10	1.48	0.02	2.07
Cer-NP (t15:1/24:1)	681.58	13.40	1.30	0.00	3.25	1.30	0.03	2.40
Cer-NS (d18:1/18:1)	609.53	11.60	1.32	0.00	4.08	1.02	0.10	2.01
HexCer-NDS (d18:0/24:1)	811.69	14.17	1.05	0.02	3.00	1.13	0.09	2.71
HexCer-NS (d30:1/12:0)	857.70	14.17	1.05	0.02	4.04	1.15	0.08	2.66
OxPC (18:0-20:3+2O)	889.61	8.57	1.15	0.01	0.45	1.65	0.00	0.49
OxPE (16:0-22:6+4O)	827.50	7.48	1.26	0.00	2.23	1.92	0.00	0.32
PC (16:2/17:2)	739.52	7.48	1.14	0.01	0.67	1.66	0.00	0.47

PC (18:0e/21:0)	817.70	14.51	1.11	0.02	0.78	1.78	0.00	0.61
PC (19:0/22:5)	849.62	12.01	1.42	0.00	0.54	1.63	0.00	0.71
PC (19:1/20:5)	819.59	7.96	1.39	0.00	0.48	1.77	0.00	0.37
PC (20:1/22:4)	863.64	12.59	1.28	0.00	0.16	1.01	0.11	0.35
PE (18:0/18:1)	745.56	12.94	1.35	0.00	1.55	1.99	0.00	1.65
PE (18:0/20:1)	773.59	12.63	1.13	0.01	1.36	1.88	0.00	1.56
PE (18:2e/24:4)	805.60	13.02	1.32	0.00	2.05	1.21	0.08	2.07
PE (19:0/20:4)	781.56	12.30	1.47	0.00	0.31	1.66	0.00	0.54
PE (20:0/20:1)	801.63	13.62	1.42	0.00	0.50	1.81	0.00	1.48
PE (20:0/20:3)	797.60	13.48	1.28	0.00	0.56	1.79	0.00	1.67
PE (21:2/20:5)	803.54	12.29	1.43	0.00	0.34	1.72	0.00	0.50
PG (16:0/20:3)	772.53	10.15	1.22	0.00	1.39	1.84	0.00	2.03
PG (20:4/22:6)	842.51	8.33	1.35	0.00	2.70	1.56	0.01	2.08
PI (16:1/18:2)	832.51	8.94	1.08	0.01	0.24	1.09	0.16	2.03
SM (d14:0/29:0)	876.72	14.98	1.29	0.00	1.54	1.90	0.00	0.52
SM (d27:0/12:1)	772.64	13.11	1.46	0.00	0.40	1.74	0.00	0.63
SM (d28:1/12:0)	786.66	13.62	1.42	0.00	0.63	1.73	0.00	0.67
TAG (15:2-18:1-18:2)	855.72	15.66	1.14	0.01	1.57	1.67	0.00	1.50

TAG (16:0-18:0-22:6)	928.75	16.97	1.48	0.00	2.75	1.79	0.00	0.62
TAG (16:0-18:2-20:3)	902.73	16.68	1.28	0.00	0.70	1.87	0.00	0.48
TAG (16:0-18:2-22:5)	921.78	16.39	1.46	0.00	1.68	1.68	0.00	0.81
TAG (18:0-18:1-18:1)	903.82	17.41	1.17	0.01	1.28	1.84	0.00	1.45
TAG (18:0-18:2-22:6)	947.79	16.60	1.45	0.00	1.79	1.67	0.00	1.39
TAG (18:1-18:2-22:5)	952.75	16.37	1.36	0.00	2.03	1.72	0.00	0.64
TAG (18:1-19:1-20:3)	939.82	17.02	1.25	0.00	1.66	1.61	0.00	1.40
TAG (18:1-20:1-22:5)	977.84	16.99	1.52	0.00	2.50	1.59	0.00	1.45
TAG (18:1-22:4-22:6)	1002.76	16.37	1.38	0.00	1.93	1.70	0.00	0.55
